# SEOM Clinical Guideline of localized rectal cancer (2016)

**DOI:** 10.1007/s12094-016-1591-0

**Published:** 2016-11-30

**Authors:** E. González-Flores, F. Losa, C. Pericay, E. Polo, S. Roselló, M. J. Safont, R. Vera, J. Aparicio, M. T. Cano, C. Fernández-Martos

**Affiliations:** 1 Complejo Hospitalario de Granada, Granada, Spain; 2 Hospital Sant Joan Despí-Moises Broggi-ICO Hospitalet, Barcelona, Spain; 3 Hospital Parc Taulí de Sabadell, Barcelona, Spain; 4 Hospital Miguel Servet, Zaragossa, Spain; 5 Hospital Clínico Universitario de Valencia, Valencia, Spain; 6 Hospital General de Valencia, Valencia, Spain; 7 Complejo Hospitalario de Navarra, Pamplona, Spain; 8 Hospital Universitario La Fe de Valencia, Valencia, Spain; 9 Hospital Reina Sofía de Córdoba, Córdoba, Spain; 10 Fundación Instituto Valenciano de Oncología, C/. Profesor Beltrán Báguena, no. 8, 46009 Valencia, Spain

**Keywords:** Rectal cancer, Treatment, Diagnostic, Guidelines

## Abstract

Localized rectal adenocarcinoma is a heterogeneous disease and current treatment recommendations are based on a preoperative multidisciplinary evaluation. High-resolution magnetic resonance imaging and endoscopic ultrasound are complementary to do a locoregional accurate staging. Surgery remains the mainstay of treatment and preoperative therapies with chemoradiation (CRT) or short-course radiation (SCRT) must be considered in more locally advanced cases. Novel strategies with induction chemotherapy alone or preceding or after CRT (SCRT) and surgery are in development.

## Introduction

Colorectal cancer represents a major health problem in developed countries. In Spain, 32,240 new cases were diagnosed (15% of global cancer incidence) and 14,700 patients died (14% of total cancer mortality) in 2012. Approximately 35% of these tumors arise in the rectum. The incidence increases with age. Median age at diagnosis is about 70 years. Other risk factors include diet (red or processed meat excess), tobacco and alcohol. Type II diabetes, ulcerative colitis and Crohn’s disease also increase the risk. The hereditary component is less pronounced for rectal cancer than for colon cancer. The Spanish Society of Medical Oncology (SEOM) invited ten experts based on major scientific contribution in the field. The purpose of this paper was to define current “state of the art” using the methodology of evidence-based medicine. The available medical literature was reviewed according to main topics of disease management, and classified by scientific levels of evidence and grades of clinical recommendation (Table 1) [1]. The resulting text was reviewed, discussed, and approved by all authors.

**Table 1:** Levels of evidence and grades of recommendation according to Infectious Diseases Society of America grading system [1]

*Levels of evidence:*
I. Evidence from at least one large randomized, controlled trial of good methodological quality (low potential for bias) or meta-analyses of well-conducted randomized trials without heterogeneity
II. Small randomized trials or large randomized trials with a suspicion of bias (lower methodological quality) or meta-analyses of such trials or of trials with demonstrated heterogeneity
III. Prospective cohort studies
IV. Retrospective cohort studies or case–control studies
V. Studies without control group, case reports, experts opinions
*Grades of recommendation*
A. Strong evidence for efficacy with a substantial clinical benefit, strongly recommended
B. Strong or moderate evidence for efficacy but with a limited clinical benefit, generally recommended
C. Insufficient evidence for efficacy or benefit does not outweigh the risk or the disadvantages (adverse events, costs, etc.), optional
D. Moderate evidence against efficacy or for adverse outcome, generally not recommended
E. Strong evidence against efficacy or for adverse outcome, never recommended

### Diagnosis

Although clear definition of rectal cancer is lacking, it is usually defined as a tumor with its lower edge within 12–15 cm from the anal verge measured by rigid sigmoidoscopy or MRI.

Adenocarcinoma is the most common histology (95–98%), other unusual tumors as melanomas, gastrointestinal stromal tumors or neuroendocrine tumors are not included in this guideline.

### Staging

Clinical stage is critical to stablish locoregional extension of the disease and risk of recurrence to individualize neoadjuvant treatment strategies in a multidisciplinary team. It requires a physical examination, a complete blood count, liver and renal function test, and carcinoembryonic antigen. A total colonoscopy to evaluate for synchronous lesions and a rigid sigmoidoscopy to determinate the location of the tumor must be performed. If the tumor is obstructive, virtual colonoscopy is recommended (but complete colonoscopy should be performed after surgery).

Both endorectal ultrasound (ERUS) and rectal magnetic resonance imaging (MRI) are recommended for early rectal cancer staging (cT1–T2), and high-resolution MRI has become the standard method for evaluating locally advanced disease particularly those patients with potential CRM involvement [2, 3]. MRI-based pretherapeutic definition of an involved CRM is an independent prognostic factor for 5-year overall survival, disease-free survival and for local recurrence [4]. MRI-defined extramural venous invasion (EMVI) is an additional independent poor prognostic factor for both local recurrence and for DFS in stage II/III rectal cancer [5]. A reliable assessment of the pretherapeutic mesorectal regional lymph node status is not possible by the present imaging modalities (Table 2 shows comparison between imaging methods.) Although surgery is the standard of care after neoadjuvant treatment, identification of complete response is interesting to detect patients who could potentially undergo organ-preserving treatment. Post neoadjuvant treatment MRI is the most studied method. The MERCURY study validated the use of high-resolution MRI for posttreatment staging and its correlation with survival outcomes (modified Mandard grading system) [6]. Some groups suggest using diffusion-weighted (DW) MRI to increase accuracy of tumor response assessment [7].

**Table 2:** Comparison between imaging methods

	ERUS	CT scan	HR MRI
T stage (early tumors)	+ Small T1–2 tumors	–	+ Assesses the depth of spread accurately to within 1 mm of histopathology assessments
T stage (advanced tumors)	–	+	+ T3 substaging (a–b/c–d)
CRM status	–	–	+ (≤1 mm from tumor to the MRF is considered as positive)
EMVI status	–	+	+
N stage	± (only perirectal lymph nodes)	–	±

Contrast-enhanced computed tomography (CT) scan of the chest, abdomen and pelvic should be carried out to estimate distant spread of the disease. Chest X-ray and abdominal MRI should be considered if CT is contraindicated.

The 7th edition of the TNM staging system should be used for clinical and histopathological staging. T1 tumors could also be classified according to Haggitt and Kikuchi stages depending on polyp morphology.

Pathological staging should include the quality of mesorectum, proximal, distal and circumferential margins (a positive CMR is defined as tumor within 1 mm from the margin), lymphovascular and nerve invasion, number of regional lymph nodes and extranodal tumor deposits and effect of neoadjuvant treatment.

## Management of local disease

### Early rectal cancer: cT1/cT2 and cN0

Oncologic outcome after rectal resection for early rectal cancer (T1–2, N0) has been proved good. Nevertheless, radical rectal surgery (RRS) implies a temporary or definitive stoma, low anterior resection syndrome and sexual and urinary dysfunction. That is why some new approaches have been proposed.

Transanal endoscopic microsurgery (TEM) has shown its superiority over local resection [8]. Two meta-analyses have compared the efficacy of TEM and total mesorectal excision for the treatment of T1 rectal cancer. Although there were not differences in distant metastases rates and overall survival, a higher risk of local recurrence was evidenced [9, 10]. Similarly, data from Norwegian Colorectal Cancer Registry show the absence of differences in 5-year relative survival rate but a significant lower 5-year local recurrence rate after total mesorectal excision [11].

Due to high local recurrence rates, TEM is not a proper technique for all patients with T1 rectal cancer but is useful for patients with low-risk T1 tumors (low tumor grade, absence of lymphovascular invasion and clear margin of resection) [12].

For T2N0 rectal cancer, total mesorectal excision is the standard of care. TEM exclusive treatment leads to inacceptable local recurrence rates. However, some alternative has been proposed in two recent trials. In phase II trial ACOSOG Z6041, selected T2N0 rectal tumors received radiotherapy combined with capecitabine plus oxaliplatin followed by local excision. After a mean follow-up of 56 months, local recurrence rate was 4%. 3- and 5-year disease-free survival for the per-protocol group was 86.9 and 80.3% [13]. These findings suggest that neoadjuvant chemoradiotherapy plus local TEM might be an alternative to radical surgery in selected patients with distal rectal cancer seeking organ-preserving surgery to avoid a permanent stoma.

### Resectable locally advanced rectal cancer: cT3–T4 any cN+

#### Surgery: open and laparoscopic

Laparoscopic surgery was first considered in 1990 for patients undergoing colectomy for cancer. Based on reports of tumor recurrences within surgical wounds, there was a concern that this approach would compromise oncologic outcome by failing to achieve a proper oncologic resection or by altering patterns of recurrence. Since then, some trials have been carried out to clear up this issue.

In rectal cancer, results of randomized trial COLOR II showed a 3-year locoregional recurrence rate of 5.0% in the two, a disease-free survival rates of 74.8% in the laparoscopic-surgery group and 70.8% in the open-surgery group, as well as absence of differences in overall survival rates (86.7 versus 83.6%) [14]. Nowadays, there was no doubt regarding the benefit in postoperative recovery of the laparoscopic approach for colorectal cancer and its equivalence in oncologic outcome.

#### Preoperative chemoradiation (CRT) or short-course radiotherapy (SCRT)

Two different preoperative approaches have been established for cStage II and III patients.

One is preoperative chemoradiation (CRT) because the german study demonstrated less toxicity and better locoregional outcomes with preoperative versus postoperative chemoradiotherapy [15]. Two studies adding concomitant 5FU or capecitabine to preoperative radiation therapy (45–50.4 Gy in 25–28 fractions) have demonstrated better local control with increased pathological responses and lower rates of local recurrences with respect to radiation therapy alone. No significant differences were observed in overall survival in these studies [16, 17]. Concomitant capecitabine is not inferior to 5FU and is standard nowadays [18, 19]. Adding oxaliplatin or targeted drugs to fluoropyrimidines do not improve outcomes and are not recommended although in some studies higher rates of pathological responses are achieved [20, 21].

The second standard approach, preoperative short-course radiotherapy (SCRT), 25 Gy over 5 days without concomitant chemotherapy, has demonstrated decreased rate of local recurrences compared to surgery alone [22].

Two randomized studies comparing both strategies (CRT versus SCRT) do not show differences in rate of local recurrence and survival. Differences in tumor downstaging were observed in favor of CRT [23, 24], so more locally advanced rectal cancers (T4/mesorectal fascia affected or close) should be treated with preoperative chemoradiation.

#### Total neoadjuvant treatment (Induction chemotherapy followed by CRT)

Induction CT may be associated with better treatment compliance and may allow full systemic doses of chemotherapy to be delivered. The timeline in the current standard treatment sequence means that full systemic chemotherapy is not delivered until about 4 months after neoadjuvant CRT is initiated.

The PAN-EX analysis showed that intensification of systemic therapy with neoadjuvant combination CT before standard treatment was feasible in poor-risk potentially operable rectal cancer, with acceptable safety and promising outcomes [25].

The Spanish GCR-3 phase II randomized trial compared preoperative CRT, followed by surgery and adjuvant CT with induction CT followed by CRT and surgery. There were no significant differences between the two arms in pCR while at the same time achieving more favorable compliance and toxicity profiles with induction versus adjuvant chemotherapy. At 5 years, there were no differences in cumulative incidence of local relapse, incidence of distant metastases DFS or OS [26].

Maréchal et al. [27] also compared standard therapy with induction FOLFOX, followed by CRT and then surgery. No differences in pCR were noted. The other four studies were single-arm trials, and the pCR rates ranged from 20% [28, 29] to 33% (Table 3).

**Table 3:** Resectable locally advanced rectal cancer: cT3–T4 any cN+: total neoadjuvant treatment (induction chemotherapy followed by CRT)

Study	No. of Pt	Induction chemotherapy	CRT regimen	Adjuvant chemotherapy	pCR (%)	Outcomes
GCR-3 [26]	108	–	CAPOX	CAPOX × 4 cy	13	5-year DFS = 62% 5-year OS = 77%
		CAPOX × 4 cy	CAPOX	–	14	5-year DFS = 64% 5-year OS = 74%
Maréchal [27]	57	–	5FU	–	34	Closed prematurely
		FOLFOX × 2 cy	5FU	–	32	
EXPERT [25]	105	CAPOX × 12wks	Capecitabine	Capecitabine × 12 wks	20	3-year DFS = 68% 3-year OS = 83%
CONTRE [28]	39	FOLFOX × 8 cy	Capecitabine	–	33	R0 = 100%
Schou [29]	85	CAPOX × 2 cy	Capecitabine	–	23	5-year DFS = 63% 5-year OS = 67%

*CRT* chemoradiotherapy, *5-FU* fluorouracil, *CAPOX* capecitabine + oxaliplatin, *XELOX* capecitabine + oxaliplatin, *FOLFOX* fluorouracil + leucovorin + oxaliplatin, *DFS* disease-free survival, *OS* Overall survival, *pCR* pathologic complete response, *pt* patient, *cy* cycle, *wk* week

**Table 4:** Recommended schedule of surveillance for rectal cancer

Office visit and CEA	Every 3–6 months for 2 years, then every 6 months until 5 years
CT chest/abdomen/pelvis	Annually for 5 years^a^
Colonoscopy	1 year after preoperative colonoscopy (or 3–6 months after surgery if colon not preoperatively “cleared”)^b^
Proctoscopy (± ERUS)	Every 6–12 months^c^ for patients who underwent resection with anastomosis or every 6 months for patients undergoing local excision for 3–5 years

*CT* computed tomography, *ERUS* endorectal ultrasound

^a^More frequent imaging may be considered for patients at particularly high risk for recurrence, including those with N2 disease, previous liver resection for metastasis, etc

^b^Further colonoscopy frequency depends on the results of the 1-year colonoscopy, with repeat examination in 3 years for patients without adenomas and 1 year for patients with adenomas. Annual colonoscopy is generally recommended for patients with confirmed or suspected familial cancer syndromes that have not undergone total proctocolectomy

^c^Patients at higher risk for local recurrence may be considered for the more frequent intervals, and for ERUS in addition to proctoscopy. Higher-risk patients may include those with poorer-risk tumors (e.g., T2 or poor differentiation) who underwent local excision, those with positive margins (≤1 mm), and those with T4 or N2 rectal cancers

Other potential advantages of the total neoadjuvant approach are: rapid relief of symptom such as rectal pain or bleeding and substantially shorter time to temporary ostomy reversal. A limitation of the induction strategy is overtreatment. So preoperative high-resolution MRI is mandatory in the selection of patients. In summary, the use of induction chemotherapy prior to neoadjuvant CRT in patients with rectal cancer can be proposed as an optional alternative in high-risk patients, but more data are needed on the use of induction strategies to know the impact on survival outcomes.

#### Adjuvant therapy

The administration of systemic adjuvant chemotherapy after preoperative chemoradiation and total mesorectal excision is controversial. Four randomized trials specifically addressing its benefit have faced with considerable challenges (old 5-FU-based schemes, poor patient accrual and compliance) and have not demonstrated its superiority over observation [30–33]. Notwithstanding this paucity of evidence, two randomized studies have compared oxaliplatin with nonoxaliplatin-containing regimens for adjuvant therapy after chemoradiation and surgery of rectal cancer [34, 21]. These two trials found moderate (4.7% and 8.7%) increase in 3-year DFS. In practice, adjuvant chemotherapy has become a widespread practice based mainly on extrapolation from results in colon cancer.

##### Are there any subgroups who may benefit from adjuvant chemotherapy?

Several studies have suggested that not all patients with rectal cancer benefit from adjuvant chemotherapy. The degree of bowel wall penetration and nodal involvement at pathology examination has been shown to be the most important predictors of long-term outcome. There is compelling evidence that patients downstaged to ypT0–2N0 after chemoradiation have very favorable prognoses, particularly in the setting of high-quality preoperative imaging and surgical technique, even in the absence of systemic treatment [35]. Even though patients with pCR seem likely to have a better outcome than do those without pCR, whether improvement in pCR in clinical trials is a useful means to select treatments that are likely to result in better survivals is unclear [36]. In contrast, node positive patients carry on significantly worse prognoses as in colon cancer.

*Recommendation* For patients treated with preoperative chemoradiotherapy, decisions about postoperative systemic treatment could be tailored to the risk of metastatic disease (based on pathology), taking also into account patient’s comorbidities, life expectancy, and preferences. They should be informed about the uncertain benefit of adjuvant chemotherapy (II, B):

(A) ypT0N0 patients must be informed about their good intrinsic prognosis and the possibility to be followed up without any more treatment.
(B) ypT1–2N0 patients can be treated with infusional 5-FU, capecitabine monotherapy or standard oxaliplatin-based chemotherapy (to complete 6 months of treatment).
(C) Most patients with ypT3-4 or N + should be offered oxaliplatin-based chemotherapy (FOLFOX × 8 or XELOX × 4–5 courses).
(D) for patients who are frail, with significant comorbidities, or with life expectancy of less than 5 years, chemotherapy should be omitted.

##### Adjuvant treatment after immediate radical standard surgery (TME)

Postoperative chemoradiotherapy with concomitant fluoropyrimidine-based chemotherapy is no longer recommended. However, it could be used in patients with positive CRM, perforation in the tumor area, defects in the mesorectum, or in other cases with high risk of local recurrence. As in stage III and high-risk colon cancer, adjuvant chemotherapy can be given, even though the level of evidence for benefit is lower in rectal cancer.

### Unresectable disease: cT4

Unresectable rectal cancer constitutes 10–15% of primary rectal cancers, due to high risk of non-radical surgery or local failure. Without an uniform definition of unresectable rectal cancer, one of these would be a tumor palpably fixed to adjacent non-resectable structures such as the proximal sacrum, pelvic sidewall, pelvic floor, prostate, or base of the urinary bladder. But the use of radiological MRI-based criteria is recommended.

In the non-resectable cases, preoperative ChemoRT was used early to sterilize peripherally located disease and induce tumor shrinkage to allow subsequent radical surgery. Fluoropyrimidines as a chemotherapy and total doses of 50.4 Gy are the standard. Some studies with 66 in 2 Gy fractions or more without compromising organs at risk have been published [37].

At the moment, there is one randomized trial comparing RT versus ChemoRT (fluoropyrimidines), and the results are significative in favor ChemoRT [38]. The results adding oxaliplatin at the treatment schedule were disappointing, and oxaliplatin is used only in some cases in the induction setting [39].

*Recommendations* Chemo/RT (Capecitabine/long-course RT or infusional 5-FU/long-course RT) or induction chemotherapy (FOLFOX or CapeOx or 5-FU/leucovorin or capecitabine) followed by CRT.

When surgery is performed without an effective result, active chemotherapy regimen for advanced disease is mandatory.

#### Follow-up

Despite potentially curative surgery including total mesorectal excision and the use of modern neoadjuvant chemoradiotherapy and adjuvant chemotherapy and/or radiation therapy (RT), about 30% of patients who present stage II or III disease will have a disease recurrence following primary therapy.

Intensive follow-up strategies are associated with a shorter time in detecting recurrences, increase the detection of asymptomatic recurrences and the proportion of patients who are potentially eligible for curative therapy. A survival benefit from such an approach has in fact been shown in several meta-analyses. According to those meta-analyses, prospective randomized clinical studies and most international groups, we recommend intensive postoperative surveillance for stage II–III patients who have undergone a curative resection of rectal cancer with or without neoadjuvant chemoradiation therapy [40, 41].

*Recommendation* (Table 4)

- A clinical encounter with a physician every 3 to 6 months for the first 3 years, and every 6 months during years 4 and 5.
- Serum carcinoembryonic antigen (CEA) level at each follow-up visit for at least the first 3 years.
- Annual computed tomography of the chest, abdomen, and pelvis for 5 years.
- Full colonoscopy one year after surgery to exclude new lesions, or 3–6 months after surgery if colon not preoperatively complete studied. Further colonoscopy frequency depends on the results of the 1-year colonoscopy, with repeat examination in 3 years for patients without adenomas and 1 year for patients with adenomas. Patients at higher risk for local recurrence may be considered for proctosigmoidoscopy every six months for three to five years. Higher-risk patients may include those who have undergone low anterior resection and not received pelvic radiation therapy, those with poorer-risk tumors (T2 or poor differentiation) who underwent local excision, those with positive margins (≤1 mm), and those with T4 or N2 rectal cancers.

## Management of local recurrence

Despite improvements in preoperative selection better treatment strategies and optimal surgery, about 10% of patients with rectal cancer will develop locoregional relapse. The management of these patients is particularly challenging.

Nearly half of locally recurrent rectal cancers (LRRC) are located in the pelvis without distant metastasis. The best treatment for LRRC in this setting is a complete resection of the recurrent tumor [42].

However, the majority of patients develop recurrence involving the pelvic wall. Extended surgery such as abdomino-sacral resection has not been popular because 5-year survival rates are low and significant postoperative morbidity. In these patients, multimodality therapy including radical surgery and chemotherapy, radiotherapy or intraoperative radiotherapy (IORT) have been reported with 5-year survival of up to 31% and local control rates of 50–71% [43, 44].

For patients with non-fixed recurrent tumors who had not received previously radiation therapy (RT), appropriate treatment would a preoperative CRT approach followed by surgical intervention. If the tumor is fixed to adjacent structures, then preoperative treatment including induction chemotherapy, chemoradiation followed by re-evaluation for surgery is the most appropriate treatment option [45, 46].

For patients with locally recurrent rectal cancer following high-dose pelvic radiation, management decisions have generally been directed toward palliative care or chemotherapy alone. Although historically considered unsafe, reirradiation in the pelvis has been investigated, and today in patients with recurrent rectal cancer who have received prior RT, the treatment with reduced-dose RT, either given daily or hyperfractionated, combined or not with chemotherapy and re-evaluation for resection could be an option. Many studies have reported until 40% 5-year overall survival rate, 60% overall response rate and 93% clinical response with a good or complete palliative effect on intractable pain and/or bleeding. The incidence of RTOG grade 3–4 toxicity was less than 10–23% [47].

Finally, intraoperative radiotherapy (IORT), either with electron beam or high-dose-rate brachytherapy, or stereotactic body radiotherapy (SBRT), could be used in selected centers with the appropriate experience with promising results.

## Special situations

### Rectal cancer and synchronous metastatic disease

Treatment strategy for synchronous oligometastatic rectal cancer should be based on the possibility of achieving R0 resection, either initially or after induction treatment for systemic disease and primary tumor.

#### Resectable

In locally advanced primary tumors (≥T3 or N+): local radiotherapy (short course) before of after preoperative oxaliplatin-based chemotherapy in combination with fluoropyrimidine (FOLFOX/CAPOX) for 3 months followed by resection of the primary (staged or synchronous) followed by postoperative FOLFOX/CAPOX for 3 months should be applied (7) [V, B].

In early primary tumors (<T3 N0): resection of primary and metastases followed by postoperative treatment with FOLFOX/CAPOX for a total of 6 months could be considered, and if necessary (e.g., CRM+, etc.) postoperative local treatment according to stage [V, B].

#### Unresectable

For initial unresectable metastatic disease, most active available induction systemic treatment should be chosen [IV, A]. If metastases become resectable, local treatment according to stage for primary followed by resection of primary and metastases should be performed, followed by postoperative continuation of the same regimen for a total of 6 months (including preoperative) [IV, A]. If metastases remain unresectable, treatment should be continued or switched, depending on the quality of response [V, B].

Never resectable metastatic disease: Treatment aim is palliation and chemotherapy should be chosen. Radical and mutilating surgery of the primary should be avoided, unless necessitated by an emergency situation [V, B].

In case of symptomatic primary of the rectum: local measures (e.g., insertion of a stent or stoma, radiotherapy) should be performed initially, and palliative surgical resection only in specific circumstances [V, B].

## Future trends: preoperative chemotherapy only/consolidative CT after CRT or SCRT

Preoperative CT without RT is attractive because pre-operative pelvic radiation is associated with an increased incidence of serious late side effects, and data from retrospective analyses suggest that radiation may not be needed in some patients with T3 tumors. Two phase II trials in T3, middle third tumors have reported encouraging results (20–27% pCR; 100% R0 resections) with an induction combination of fluoropyrimidine/oxaliplatin and bevacizumab [48, 49]. In a similar population the phase II/III intergroup Prospect trial (NCT01515787) is now open and compares pre-operative chemotherapy with selective chemoradiation versus standard pre-operative CRT in a similar population.

Tumor response to RT is time-dependent, and longer intervals from RT to surgery and delivering systemic chemotherapy after chemoradiation rather than before might have a greater effect on tumor response [50]. In a non-randomized phase 2 trial with four sequential groups of patients with rectal cancer, adding up to six cycles of modified FOLFOX6 (mFOLFOX6) CT between preoperative CRT and surgery, increased the proportion of patients who achieved a pCR [51].

This strategy is being compared with induction CT followed by CRT and surgery in a randomized multi-institutional American trial. NCT02008656 [52].

A randomized phase 3 trial comparing SCRT followed by consolidation CT with FOLFOX4 versus long-course CRT, showed an improvement in 3-year overall survival with the first approach, without differences in pCR rate, local control or disease-free survival [53]. The acute toxicity was significantly lower in the SCRT arm. Another trial (RAPIDO) compares short-course RT followed by prolonged preoperative CT and surgery with standard CRT and surgery and optional adjuvant CT in high-risk rectal cancer [54].
